# Evaluation of prothymosin alpha, trimethylamine-N-oxide, and ischemia-modified albumin in type 2 diabetes mellitus patients with dysregulated lipid profile

**DOI:** 10.5339/qmj.2025.39

**Published:** 2025-06-11

**Authors:** Karam Mazin Gharab, Mohammad Ahmad Bik, Safaa Ehssan Atta, Ghufran S. Jawad, Eissa Almaghrebi, Isam Noori Salman, AIi Unlu

**Affiliations:** 1 National Diabetes Center, Mustansiriyah University, Baghdad, Iraq; 2 Medical Biochemistry Department, Faculty of Medicine, Selcuk University, Konya, Turkey *Email: ghufransalman@uomustansiriyah.edu.iq

**Keywords:** Diabetes mellitus, hyperlipidemia, ischemia-modified albumin, prothymosin alpha, trimethylamine-N-oxide

## Abstract

**Background:** Prothymosin alpha (PTMα) is a small acidic polypeptide from the thymosin family with immune activity and protective properties against oxidative stress induced by reactive oxygen species trimethylamine-N-oxide (TMAO), produced in the liver from gut bacterial metabolite trimethylamine and associated with increased cardiovascular disease risk and higher all-cause mortality. Ischemia-modified albumin (IMA) is a significant oxidative stress biomarker, particularly in ischemia-reperfusion conditions. This study investigates PTMα, TMAO, and IMA levels in type 2 diabetes mellitus (T2DM) patients, both with and without hyperlipidemia, to explore their relationships and their potential role as biomarkers or therapeutic targets.

**Method:** The study received ethical approval from the Selcuk University Faculty of Medicine Hospital committee under approval number 2024/33. The study included male and female T2DM patients aged 30–60, with 30 having hyperlipidemia and the rest being non-lipemic. TMAO was performed using API 3200 LC-MS\MS while PTMα was analyzed using an ELISA kit from BT LAB, serum IMA levels were evaluated by the spectrophotometric method.

**Results:** Comparisons were made between those with T2DM and control groups. In the T2DM group, PTMα was significantly higher in females (*p* = 0.047), while TMAO and IMA showed no significant gender difference. The control group had no significant differences in PTMα, TMAO, and IMA levels. Comparisons among healthy controls, non-lipemic T2DM patients, and hyperlipidemic T2DM patients revealed significantly decreased PTMα levels with no change in IMA levels across groups. In contrast, TMAO was significantly higher in the patient group.

**Conclusion:** The findings of this study have potential implications for the field, suggesting that PTMα might serve as a prognostic indicator for T2DM and that reduced TMAO levels might play a role in T2DM pathogenesis, opening up new avenues for research and treatment.

## INTRODUCTION

Diabetes mellitus (DM) is a chronic metabolic condition characterized by persistent high blood glucose. The condition may result from insufficient insulin production by pancreatic β-cells, insulin-resistant tissues, or both. Continued high blood glucose levels, in addition to other metabolic imbalances in individuals with diabetes, can damage different organ systems, resulting in the onset of severe and life-threatening health complications. People with DM may develop complications that include both macrovascular and microvascular conditions. These macro cardiovascular issues include heart disease, strokes, and peripheral artery disease, while microvascular conditions such as kidney disease are linked to diabetes, retinopathy, and peripheral neuropathy.^1–4^

Insulin resistance is a state of decreased responsiveness to high physiological insulin levels in physiologically insulin-targeting tissues. It is considered a pathogenic driver of many modern diseases, including metabolic syndrome, metabolic dysfunction-associated steatotic liver disease, atherosclerosis, and type 2 diabetes mellitus (T2DM).^5^

Hyperlipidemia is a systemic metabolic disease defined by high blood lipids, including triglycerides and cholesterol. It has been linked to a variety of health issues, including metabolic syndrome, a combination of diabetes, obesity, and hypertension, that cause significant risks to human health.^6^

Prothymosin alpha (PTMα) is a small acidic polypeptide with immune activity that belongs to the thymosin family. Encoded by the PTMA gene in humans (2q37.1), It is composed of 100–109 amino acids and was first identified in the thymus, although it is also expressed in several types of mammalian cells.^7^ Despite being described in 1984, the biological effects of this protein have been a topic of controversy. Initially, PTMα was thought to be a thymic factor with a hormonal-like role in T-lymphocyte maturation. Physiologically, PTMα, a nuclear polypeptide, has a nuclear localization signal and is not secreted because it lacks the necessary signal peptide sequence for secretion.^8^

PTMα has been found to safeguard cells from oxidative stress induced by reactive oxygen species (ROS). This unstable molecule contains oxygen that readily interacts with other molecules within a cell. Moreover, it shields cells from apoptosis by prolonging the apoptotic cascade or by inhibiting apoptosome formation.^7^ PTMα can activate neutrophils in cancer patients, boosting their phagocytic capacity and ROS production, thereby increasing their ability to destroy cancer cells.^9^ PTMα is also utilized as an indicator for proliferation and is associated with tumor intensity in various types of cancer.^10^

Recent research indicates that PTMα, a protein, has been found to modulate immune responses, oxidative stress, proliferation/apoptosis, obesity, neuroprotection, and cancer.^11^ It also plays a crucial role in insulin resistance and fibrosis.^12^ A recent study showed that PTMα has potential as a diagnostic marker and/or therapeutic agent.^13^

The study showed that transgenic mice overexpressing PTMα become insulin-resistant. In addition, using a lentiviral vector to inhibit PTMα in mice on a high-fat diet can help prevent this condition. The study also found higher levels of serum PTMα in patients with T2DM compared to control subjects, even after adjusting for body mass index.^14^

In another study, the connection between G-protein inhibitory (Gi) and Phospholipase C (PLC) was shown via a reconstitution experiment. Within this experiment, it was observed that PLC activation mediated by Gi-coupled receptors in the brain membrane (resulting in Ins1,4,5-P3 production or Ca^2+^ movement), which was eliminated with the application of pertussis toxin, could be reinstated by adding back purified Gi1, while Go had no such effect. Based on this, the researchers hypothesize that PTMα plays a role in preventing cell death through necrosis by restoring the flow of glucose into cells and maintaining cellular ATP levels. The process involves the movement of glucose transporters 1–4 (GLUT1/4) to the cell surface, which is part of the signaling pathways activated by the possible interaction with a Gi-coupled PTMα receptor, leading to the activation of PLC and PKCβ2.^15^

Obesity is linked to a chronic, low-grade inflammatory condition, which increases the likelihood of insulin resistance and issues related to heart and metabolic health. A significant feature of visceral fat in people with obesity is hypoxia, which results in changes to the secretions of 3T3-L1 adipose cells in mice. This leads to an increase in the levels of PTMα, a protein first identified in the thymus and known for its role in modulating the immune system.^11^

Since the studies showed that PTMα might be related to insulin resistance, they also showed that obesity upregulates PTMα. Hence, this study aims to investigate the levels of PTMα in diabetes patients with and without hyperlipidemia to seek a relationship and the potential role of PTMα as a biomarker or therapeutic target.

Trimethylamine-N-oxide (TMAO) is produced in the liver through the oxidation of gut bacterial metabolite trimethylamine (TMA). TMA is synthesized from carnitine and choline with the assistance of enzymes derived from the CutC and CntA genes, respectively.^16^ Multiple pieces of evidence indicate a link between elevated levels of TMAO and an increased risk of cardiovascular disease and higher all-cause mortality.^17^ One important function of TMAO is its ability to affect the structure and activity of a wide range of biologically important compounds. TMAO plays a critical role in stabilizing the folded state of proteins and nucleic acids.^18^

Higher concentrations of TMAO have been linked to impaired glucose regulation and worse clinical outcomes in diabetic complications. Notably, reducing plasma TMAO levels through FMO3 knockdown in insulin-resistant mice has been shown to prevent hyperlipidemia and hyperglycemia.^19^ This evidence suggests that TMAO could be a valuable biomarker for diabetes-related diseases, prompting our investigation into its levels in patients with DM with and without hyperlipidemia.

Albumin is a highly abundant protein in the human body, making up about 40% of the proteins in the bloodstream. Its properties change during ischemic attacks that are linked to oxidative stress, the production of ROS, and the development of acidosis.^20^

Ischemia-modified albumin (IMA) is generated in response to ischemic stress, which induces alterations in the circulating albumin molecules. The albumin molecule’s N-terminal end traditionally binds transitional metals, including cobalt, copper, and nickel. However, ischemic conditions, potentially resulting from hypoxia, acidosis, free radical damage, and disruption of energy-dependent membranes, modify the N-terminus of albumin, diminishing its metal-binding capacity.^21^

IMA is a significant biomarker of oxidative stress, especially in conditions related to ischemia-reperfusion. This includes various clinical conditions such as chronic kidney disease, hypercholes-terolemia, systemic sclerosis, and, as preliminary reports suggest, T2DM.^22,23^ This highlights the clinical importance of IMA and its potential as a diagnostic tool in these conditions.

Choosing PTMa, TMAO, and IMA as biomarkers for investigating T2DM in individuals both with and without hyperlipidemia offers a broad view of the condition. Each of these markers addresses different yet interrelated biological processes crucial for the improvement of T2DM, especially regarding inflammation, oxidative stress, and metabolic imbalance. By studying these biomarkers, researchers can achieve a deeper understanding of T2DM and its related complications.

The objective of this research is to examine the interconnectedness among three significant biomarkers—PTMα, TMAO, and IMA in individuals diagnosed with DM, with a focus on those who also suffer from hyperlipidemia. PTMα is recognized for its involvement in immune regulation, insulin resistance, and oxidative stress. On the other hand, TMAO is known for its links to cardiovascular risks, glucose regulation, and complications related to diabetes. IMA, a marker of oxidative stress and ischemic conditions, could further indicate metabolic imbalances in these patients. This study aims to explore the combined effects of certain biomarkers on insulin resistance, lipid metabolism, and oxidative stress. This could improve their potential as markers or treatment options for complications related to diabetes.

**Ethics approval:** This research received ethical clearance from the ethics board of the Selcuk University Faculty of Medicine Hospital, under the approval number 2024/33, dated Jan 16, 2024. This study was performed in line with the principles of the Declaration of Helsinki.

## PATIENTS AND METHODS

Serum samples of 60 patients and 30 controls were collected from T2DM patients in gel and EDTA tubes. The gel tube was centrifuged (Beckman Coulter Allegra X-22R) after clotting at 4000 g for 10 minutes before transferring to the Eppendorf tube and stored at −80°C until analysis (Sanyo Electric Co. Ltd. Model: MDF-U5186S, JAPAN).

### Participant enrollment

Patients between the ages of 30 and 60, male and female, diagnosed with T2DM were included in the study. Thirty of the patients had hyperlipidemia, and the others were non-lipemic patients. Diagnosis of T2DM was done by the endocrinologist and considered when the fasting blood glucose (FBG) levels were greater than 110 mg/dL, 2-hour postprandial blood glucose was higher than 160 mg/dL, and all patients had HbA1c >6.5. The threshold value for selecting hyperlipidemia patients was cholesterol >200 mg/dL and triglyceride > 150 mg/dL.

### Study procedure

On the day of analysis, the serum samples were brought at room temperature in the biochemistry laboratory of the Selcuk University Faculty of Medicine Hospital. FBG, lipids, insulin, and were measured with the Cobas 8000. A complete blood count was measured using the Sysmex 3000 analyzer. The HbA1c value was measured with the Premier HB9210 analyzer. Homeostatic Model Assessment for Insulin Resistance (HOMA-IR) is calculated by the following formula: HOMA-IR = Glucose (mg/dL)×Insulin (μIU/mL)/405.

PTMα was analyzed using an ELISA kit from BT LAB (CAT No: E2263Hm). According to the manufacturer’s instructions, the detection range was 0.2–70 ng/mL. The average sensitivity was 0.1 ng/mL. TMAO was analyzed using previously validated API SCIEX 3200 LC-MS/MS methods. Serum IMA levels were evaluated by the spectrophotometric method as the procedure used by Onmaz and Sivrikaya (Perkin et al. 25 UV/Vis, US).^24^

### TMAO procedure

100 μL of d9-TMAO to 250 μL serum samples, then 1000 μL of methanol, were taken. After adding methanol, the samples were vortexed for 30 seconds and centrifuged at 14,000 rpm for 10 minutes. After separating the supernatant, the liquid supernatant was evaporated at 28°C under nitrogen gas. After evaporation, the samples were dissolved with 250 μL distilled water centrifuged at 4500 rpm for 10 minutes, and 250 μL of the supernatant was pipetted into HPLC vials, and the analysis was performed, as described in the S¸engül study.^25^

### IMA procedure

IMA levels were measured by the method reported by Onmaz and Sivrikaya.^24^ Briefly, 200 μL serum sample and 50 μL 0.1% cobalt chloride were added to Eppendorf centrifuge tubes. The mixture was vortexed for 10 seconds and then incubated at room temperature for 10 minutes. At the end of the time, 50 μL of dithiothreitol (DTT) (1.5 mg/mL) was added to the reaction mixture to enable the colorimetric reaction with free cobalt and incubated at room temperature for 2 minutes. After incubation, 1 mL isotonic saline solution was added to the mixtures in Eppendorf centrifuge tubes to stop the reaction. The blind tube was prepared for each serum sample using the same procedure without the addition of DTT. The absorbance values of the samples and the blinds were measured on a spectrophotometer (PerkinElmer Lambda 25 UV/Vis, US) set at 470 nm wavelength, and the difference between them was expressed as serum IMA levels.

### Inclusion criteria

- Subjects with confirmed diagnosis of T2DM
- HbA1c levels above 6.5%
- FBG levels are more than 110 mg/dL
- Having a stable health condition
- Presence of normal kidney function
- Being 18 years or older

### Exclusion criteria

- Presence of other types of diabetes
- History of major surgery
- Pregnancy or lactation
- Chronic liver or kidney disease
- Substance abuse or alcoholism
- Severe cardiovasculardisease

### Statistical analysis

Statistical data were analyzed using the Statistical Package for the Social Sciences (SPSS) version 26.0 (SPSS Inc, Chicago, IL, USA) software program. Kruskal-Wallis H test was used to evaluate the level of significance between the patients and control groups. Results are presented as median, minimum, and maximum. Spearman test was used for correlation. A result was considered statistically significant if the p value was below 0.05.

## RESULTS

Sixty DM patients and 30 control subjects were included in this study. Half of the patients had hyperlipidemia, while the others had average lipid profiles. The subjects’ ages were between 30 and 60, with a mean of 46.10 ± 16. Patients’ groups had higher TG, CH, and LDL levels than the control group (*p* = 0.000, *p* = 0.000, and *p* = 0.000, respectively), while HDL showed no difference between patients and controls (*p* = 0.492). Additionally, WBC, MCH, and MCHC were significantly higher in the DM group compared to control (*p* = 0.016, *p* = 0.005, and *p* = 0.003, respectively); on the other hand, RBC, MCV, HGB, HCT, and PLT showed no significant difference between groups (*p* = 0.232, *p* = 0.101, *p* = 0.126, *p* = 0.414 and *p* = 0.399 respectively) medians, minimum, and maximum are shown in Table 1.

PTMα, TMAO, and IMA levels in DM and control groups were compared according to sex. In the DM group, while PTMα was significantly higher in females (*p* = 0.047), TMAO and IMA showed no significant difference. PTMα, TMAO, and IMA showed no significant differences in the control group.

Biochemical parameters between control, non-lipidemic DM patients, and hyperlipidemic DM patients are shown in Table 1. The comparisons between healthy control, non-lipidemic DM patients, and hyperlipidemic DM patients reveal decreased PTMα concentrations with no change in IMA levels between groups. At the same time, TMAO was significantly higher in the patient group (*p* = 0.000). FBG, HbA1c, insulin, and HOMA-IR levels were significantly higher in hyper-lipemic and non-lipemic DM patients compared to healthy control, as shown in Table 2.

In correlation studies, PTMα showed a strong negative correlation with FBG, HbA1c, insulin, and HOMA-IR. TMAO showed a strong positive correlation with the mentioned markers above, while IMA did not show any significant correlation. PTMα also showed a negative correlation with TMAO. IMA showed no correlation with PTMα and TMAO. All correlation coefficients (r) and *p* values are shown in Table 3.

## DISCUSSION

T2DM is a chronic metabolic condition that can lead to complications affecting the kidneys, retina, heart, nerves, and liver.^26^ It is characterized by continuous low-grade inflammation, which is related to the development of insulin resistance.^27^

In diabetes, persistent high blood sugar levels and mitochondrial dysfunction can lead to raised production of ROS, which in turn boosts oxidative stress. This can have several negative effects related to diabetes, including reduced beta-cell function and increased insulin resistance. Additionally, oxidative stress can harm blood vessels, which is key to the development of diabetic complications like retinopathy, nephropathy, and cardiovascular diseases. There is a notable connection between inflammation and oxidative stress. The immune response initiates the release of proinflammatory cytokines and chemokines, which activate macrophages that produce ROS to combat pathogens. In the case of chronic inflammation related to diabetes, there can be a continuous generation of ROS. This ongoing process results in cellular harm and exhausts the body’s antioxidant resources, making it more challenging to manage oxidative stress effectively.^28^

Looking at these three biomarkers offers valuable insights into key aspects of T2DM pathology: inflammation (PTMa), oxidative stress (IMA), and metabolic dysfunction (TMAO). By combining these indicators, we get a clearer picture of the inflammatory, oxidative, and metabolic issues that are common in T2DM. This deeper understanding helps us grasp the complexities of the disease and its complications. PTMa, TMAO, and IMA together highlight how various pathways interact and influence the progression of T2DM. Such insights can lead to more targeted treatments for those living with T2DM, particularly for those also facing challenges like high cholesterol.

Recent research conducted by Greco and Mirabelli has found that ProTα may serve as a sensitive biomarker of inflammation and insulin resistance in obese individuals.^11^ Additionally, another study revealed that PTMα can activate NFκB, leading to increased translocation of NFκB to the nucleus, thereby disrupting the Akt/glycogen synthase kinase 3-β signaling pathway and inducing insulin resistance. These findings shed light on the potential pathophysiological role of PTMα and its novel mechanism in the development of diabetes. Notably, the same study showed elevated levels of PTMα in diabetic mice compared to the control group.^14^ Interestingly, our study results showed the opposite. PTMα levels were significantly lower in DM groups than in health control (*p* = 0.000). As far as we know, this is the first study to investigate PTMα levels in human serum. Also, there was a strong negative correlation between PTMα and FBG, HbA1c, Insulin, and HOMA-IR (*p* = 0.000, *p* = 0.001, *p* = 0.000, *p* = 0.000, respectively).

Research suggests that PTMα plays a role in preventing necrosis through specific mechanisms. These mechanisms include the activation of a presumed Gi-coupled receptor by PTMα, which then leads to the activation of PLC and PKC-β2. This cascade ultimately facilitates the movement of GLUT1/4 to the plasma membrane. This translocation leads to an influx of glucose, which elevates the levels of glucose within cells through the process of glycolysis. Consequently, the levels of ATP inside the cells rise, which helps to avert cell death. Low levels of PTMα, especially in patients with insulin resistance, might exacerbate the disease’s progression by exposing cells to necrosis due to insufficient ATP production. Further studies are essential to investigate the role of PTMα in patients with DM.

The proposed mechanism suggests that PTMα activation leads to increased glucose uptake and ATP production, potentially beneficial in conditions like diabetes where glucose metabolismis dysregulated. However, the results of the current study, interestingly and in contrast to the hypothesis, showed lower PTMα levels in DM. This alteration in diabetic individuals and whether these alterations contribute to disease pathology or represent a compensatory mechanism requires further investigation.

Despite the mechanism underlying the changes of PTMα, study results showed that PTMα might serve as a prognosis indicator since it had a strong negative correlation with DM biomarkers or therapeutic response. Additionally, understanding the mechanisms underlying the changes of PTMα and how it affects glucose metabolism could lead to the development of novel therapeutic strategies for managing diabetes and its complications.

TMAO is a small organic compound found in the blood, and its levels rise after consuming dietary l-carnitine and phosphatidylcholine. Recent clinical studies indicate a link between elevated TMAO levels and an increased risk of major adverse cardiovascular events, including heart attacks, strokes, and even death. There is growing evidence suggesting that high plasma TMAO levels could serve as a new marker for heightened cardiovascular risk in humans.^29^

A recent investigation revealed that higher levels of serum TMAO were linked to an increased risk of T2DM and a rise in FBG levels in middle-aged and elderly Chinese adults.^30^ Other related study results suggest an association between plasma TMAO levels and T2D. A significant correlation was found between red meat consumption and increased levels of TMAO in T2D patients.^31^ Another study showed that dietary TMAO exacerbated impaired glucose tolerance in high-fat diet-fed mice, increasing fasting insulin levels and HOMA-IR. The same study suggested that TMAO altered the expression of genes related to insulin signaling, glycogen metabolism, and glucose transport in the mice’s livers.^32^

Lever and collaborators found significantly higher median plasma TMAO concentration in diabetic patients than in the non-diabetes subjects and suggest that elevated plasma TMAO is a substantial risk marker in diabetes.^33^ The current study investigated the levels of TMAO in DM patients with hyperlipidemia and DM patients with average lipid profiles and healthy controls. Study results showed that TMAO was significantly higher in the DM group with and without hyperlipidemia than healthy controls with *p* = 0.000. The results showed no significant difference between DM with and without hyperlipidemia, reflecting the importance of this marker in both groups. Also, there was a strong positive correlation between TMAO and FBG, HbA1c, insulin, and HOMA-IR (*p* = 0.002, *p* = 0.000, *p* = 0.000, *p* = 0.000, respectively). This strong correlation indicates that similar underlying factors influence these markers, and further investigation is needed to understand these factors and how TMAO is important in DM diagnosis or even as a therapeutic target for drugs. It is essential to mention that TMAO is significantly correlated to a diet of red meat, so monitoring TMAO levels may be an assessment of the diet habits of this patient group. Further studies are needed, considering overall dietary patterns, physical activity, and medical history.

It has been reported that serum IMA levels increase in diseases involving ischemia, hypoxia, or inflammation, such as myocardial infarction, pulmonary embolism, cerebrovascular ischemic diseases, cancer, diabetes, multiple sclerosis, and psoriasis. In addition, IMA levels have been shown to increase in various inflammatory rheumatological diseases such as ankylosing spondylitis, Sjögren’s syndrome, and Behçet’s syndrome.^34^

Another study investigated IMA levels and showed a correlation between poor glycemic control and dyslipidemia in T2DM, which might serve as an indicator of oxidant stress in this patient group.^22^ Our study investigated the levels of IMA in T2DM with and without hyperlipidemia and in healthy controls. Results showed no significant difference between these groups.

Many experimental studies have shown a clear connection between oxidative stress and diabetes by assessing markers of oxidative stress in individuals with diabetes.^35^ IMA is considered an oxidative stress marker; thus, we hypothesized that IMA levels will be elevated in T2DM patients due to oxidative stress in these patients; interestingly, in our study, levels of IMA were not related to disease and did not show any significant correlation with used DM markers in this study. This difference, in contrast to past studies in the literature, might be due to genetic factors and the lifestyle of the study population or other factors such as divergent therapeutic protocols.

The relatively small sample size and single-center design may limit the generalizability of our findings. Also, the potential influence of unaccounted factors such as diet, medication, and genetic variability may affect these findings. We believe it is important to identify these limitations, as they help provide context for our findings and can guide future research in the right direction.

## CONCLUSION

This study highlights the roles of PTMα, TMAO, and IMA in T2DM. PTMα serum levels were significantly lower in T2DM patients than in healthy control, showing a strong negative correlation with glycemic markers, suggesting its potential as a prognostic marker. TMAO serum levels were elevated in T2DM patients and showed a positive correlation with markers of glucose metabolism, emphasizing its role in metabolic dysfunction and cardiovascular risk. In contrast, IMA levels did not show significant differences or correlations, indicating limited relevance in this study population. These findings underline the need for further investigation into PTMα and TMAO as biomarkers and therapeutic targets in T2DM management.

## Acknowledgments

The authors express their gratitude to Selcuk University for offering the necessary facilities for this research and acknowledge the consent of their patients to publish this study.

## Conflict of interest

The authors declare that they have no conflict of interest.

## Authors’ contribution

All authors contributed to the study’s conception and design. Material preparation, data collection, and analysis were performed by KG, MB, and EA. The first draft of the manuscript was written and edited by KG, SA, and GJ, and all authors commented on previous versions of the manuscript. Supervision was done by IS and AU. All authors read and approved the final manuscript.

## Figures and Tables

**Table 1.** Biochemical parameters within study groups.

**Metabolite**	**Control (median, min-max)**	**DM with no lipidemia (median, min-max)**	**DM with hyperlipidemia (median, min-max)**	***p* value**
TG (mg/dL)	109.5 (62–118.47)	104 (64–142)	283 (153–718)	***p* = 0,000**
CH (mg/dL)	167.8 (135–189)	165.5 (118–199)	233.5 (178–428)	***p* = 0.000**
HDL (mg/dL)	43.3 (36.6–48.2)	46.5 (30–77.8)	42.5 (23–65)	*p* = 0.492
LDL (mg/dL)	93.7 (84–119)	92.4 (43–138.2)	134 (50–325.5)	***p* = 0.000**
WBC (K/μL)	6.73 (5.27–8.44)	7.4 (4.65–14.94)	8.1 (5.4–14)	***p* = 0.016**
RBC (10e6/μL)	4.77 (4.2–5.63)	4.92 (3.82–5.95)	5.11 (3.89–5.91)	*p* = 0.232
HB (g/dL)	14.15 (12.8–16.7)	13.4 (7.5–17)	14 (11.2–17)	*p* = 0.126
HCT (%)	42.65 (38.9–47.6)	41 (27.8–51.6)	42.25 (35.4–48.7)	*p* = 0.414
MCV (fL)	87.6 (80.8–92.8)	86 (62.4–101)	85.5 (64–93)	*p* = 0.101
MCH (pg)	29.5 (27.3–31.2)	27.85 (17.3–31.8)	27.5 (19.5–31.4)	***p* = 0.005**
MCHC (g/dL)	33.7 (32.1–35.6)	32.4 (27.7–34.3)	32.55 (29.8–35.5)	***p* = 0.003**
PLT (K/μL)	265.5 (183–334)	281 (92–458)	284 (168–563)	*p* = 0.399

*p* values calculated using the Kruskal-Wallis H test. Bolded values indicate statistically significant differences (*p* < 0.05). TG: triglyceride, CH: cholesterol, HDL: high-density lipoprotein, LDL: low-density lipoprotein, WBC: white blood cell, RBC: red blood cells, Hb: hemoglobin, HCT: hematocrit, MCV: mean corpuscular volume, MCH: mean corpuscular hemoglobin, MCHC: mean corpuscular hemoglobin concentration, PLT: platelets.

**Table 2.** Medians, minimum, maximum, and p values of study parameters between patient groups and healthy control.

**Metabolite**	**Control (median, min-max)**	**DM with no lipidemia (median, min-max)**	**DM with hyperlipidemia (median, min-max)**	***p* value**
PTMα (ng/mL)	15.96 (4.94–89.92)	7.14 (4.5–40)	6.55 (2–16.36)	***p* = 0.000**
TMAO (ng/mL)	316 (67.7–1910)	656.5 (199–3350)	598 (212–4090)	***p* = 0.000**
IMA (ABSU)*	0.159 (0.11–0.277)	0.16 (0.004–0.432)	0.18 (0.014–0.463)	*p* = 0.324
FBG (mg/dL)	88 (70–99)	141.5 (69–547)	212 (94–409)	***p* = 0.000**
HbA1c (%)	5.5 (5.2–5.7)	7.65 (6.2–15)	9.05 (6.3–14.6)	***p* = 0.000**
Insulin (μIU/mL)	5.13 (1.68–9.46)	8 (0.4–58.4)	13.4 (0.4–35.3)	***p* = 0.000**
HOMA-IR	1.13 (0.33–2.05)	3 (0.1–24.7)	7.3 (0.4–24.8)	***p* = 0.000**

*p* values calculated using the Kruskal-Wallis H test, Bolded values indicate statistically significant differences (*p* < 0.05),

*Absorbent unit.

**Table 3.** Correlation results between all parameters.

**Parameters**	**ProT**		**TMAO**		**IMA**		**FBG**		**HbA1c**		**Insulin**		**HOMA-IR**	
	***r***	***p***	***r***	***p***	***r***	***p***	***r***	***p***	***r***	***p***	***r***	***p***	***r***	***p***
ProT	1000	-	−0.297	**0.004**	−0.090	0.397	−0.404	**0.000**	−0.443	**0.000**	−0.352	**0.000**	−0.422	**0.000**
TMAO	−0.297	**0.004**	1000	-	−0.080	0.452	0.316	**0.002**	0.378	**0.000**	0.415	**0.000**	0.421	**0.000**
IMA	−0.090	0397	0.080	0.452	1.000	-	0.047	0.658	0.020	0.852	0.048	0.666	0.012	0.913
FBG	−0.404	**0.000**	0.316	**0.002**	0.047	0.658	1.000	-	0.823	**0.000**	0.368	**0.001**	0.613	**0.000**
HbA1c	−0.443	**0.000**	0.378	**0.000**	0.020	0.852	0.823	**0.000**	1.000	-	0.439	**0.000**	0.633	**0.000**
Insulin	−0.352	**0.001**	0.415	**0.000**	0.048	0.666	0.368	**0.001**	0.439	**0.000**	1.000	-	0.872	**0.000**
HOMA-IR	−0.422	**0.000**	0.421	**0.000**	0.012	0.913	0.613	**0.000**	0.633	**0.000**	0.872	**0.000**	1.000	-

*r*: Spearman’s rho correlation coefficient. Bolded values indicate statistically significant differences (*p* < 0.05).
